# Highly Fluorescent Terbium-Doped Graphene Quantum Dots (Tb-GQD) for Rapid and Selective Detection of Tartrazine in Food Matrices

**DOI:** 10.1021/acsomega.6c04540

**Published:** 2026-08-03

**Authors:** Tharani Gomathi Ramesh, Mangaiyarkarasi Rajendiran

**Affiliations:** Centre for Nanotechnology Research, 30026 Vellore Institute of Technology, Vellore 632014, India

## Abstract

Terbium-doped graphene quantum dots (Tb-GQDs) were synthesized using carbonization and surface coordination and subsequently used as efficient fluorescent probes for the quantitative and selective determination of tartrazine in food samples. The doping of terbium enhanced the photostability of GQDs. Photoluminescence emission spectra revealed that the fluorescence of Tb-GQDs was effectively quenched by increasing concentrations of tartrazine, which was attributed to a combination of static quenching and the inner filter effect (IFE). Under optimized conditions, the Tb-GQD probe showed a linear response with a detection limit of 6.7 nM. Tb-GQDs functioned as effective sensor platforms for the selective detection of tartrazine. Analytical studies using commercial food samples demonstrated recovery rates ranging from 97.4 to 101.5%, confirming the reliability and practical applicability of the proposed method. Because of their straightforward synthesis, rapid response, and high sensitivity, Tb-GQDs show strong potential for real-time monitoring of synthetic food colorants such as tartrazine.

## Graphical abstract

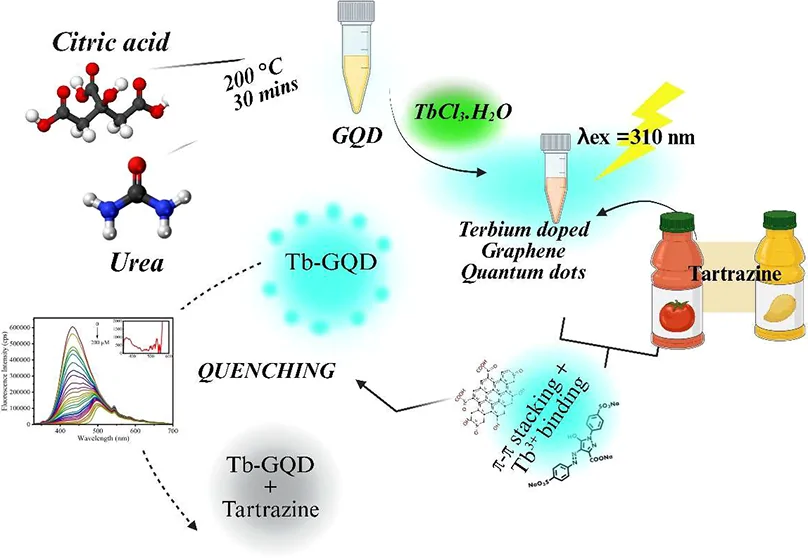

## 1 Introduction

Artificial food colors are synthetic dyes that impart intense coloration to food products, thereby enhancing their visual appearance. These dyes are available in various forms, including powders, liquids, and gels, and are commonly used in candies, baked goods, beverages, and other processed foods. Among these, tartrazine is a yellow azo dye, also known as Acid Yellow 23 or FD&C Yellow 5. The chemical name of tartrazine (Tz) is tri-sodium 5-hydroxy-1-(4-sulfonato phenylazo) pyrazole-3-carboxylate.^1,2^ According to the FSSAI amendment regulation 2020, foods and beverages may contain up to 100 mg/kg of tartrazine, with an average daily consumption of 7.5 mg/kg body weight.^3,4^ Tartrazine is readily incorporated into food products due to its water solubility. It induces oxidative stress and DNA damage in tissues, contributing to the development of cancer cells in humans.^5^ Tartrazine also elevates serum levels of cancer biomarkers such as α-fetoprotein and CA 15–3, which are associated with malignancies and tumor growth.^5^ Due to these adverse health effects, the use of tartrazine is prohibited or strictly regulated in several countries. Although permissible levels are considered safe for most individuals, frequent intake of tartrazine can be harmful to those with allergies, asthma, neurobehavioral disorders, liver disorders, or cancer.^6,7^ Therefore, sensitive analytical techniques have been employed to detect tartrazine levels in food and consumer products to prevent excessive intake. Methods such as high-performance liquid chromatography (HPLC), electrochemical analysis,^8–10^ thin-layer chromatography (TLC), liquid chromatography–mass spectrometry (LC-MS), and capillary electrophoresis have been used to detect tartrazine in food samples.^11–13^ Although these conventional methods offer high sensitivity, they are often time-consuming, costly, and require complex sample preparation. As an advancement, fluorescent-based techniques have gained significant interest due to their rapid screening, higher detection rates, greater specificity, simplified sample preparation, enhanced sensitivity and selectivity, and practical applicability for real-time detection. In this aspect, fluorescent nanomaterials emerge as efficient sensing probes for the detection of various analytes like metal ions, carcinogenic chemicals, and biomolecules.^14–17^ These materials also offer unique optical properties, improved integration with biomolecules, tunable surface characteristics, and the potential for developing portable, ultrasensitive, and cost-effective sensors.^18^

Among the various fluorescent nanomaterials, graphene quantum dots (GQDs) have drawn interest because of their excellent photostability, biocompatibility, water solubility, tunable bandgap, and optical properties, making them suitable for biosensing applications.^19^ GQDs are made of single or a few layers of graphene sheets with a size less than 10 nm. Because of its well-defined sp^2^ hybridized honeycomb lattice with quantum confinement and edge effects, it possesses stable and tunable photoluminescence properties.^20^ In addition, the surface states of GQDs are abundant in oxygen-containing functional groups, providing several anchoring sites for sensing metal ions, biomolecules, and organic analytes.^14,21–23^ Although bare GQDs have many advantages, they have certain limitations, such as less stability, lower quantum yield, susceptibility to photobleaching, and a lack of multifunctionality and luminescent tunability compared to some rare-earth elements. To overcome the above challenges, doping GQDs with rare-earth elements results in enhanced quantum yield, high stability, and the ability to sense multiple analytes.^24–28^ In particular, unique 4f electron configurations of Terbium (Tb^3+^) exhibit sharp emission, large Stokes shift, longer luminescent lifetimes, and increased photostability.^29,30^ This makes RE-doped GQD highly promising for bioimaging, sensing, the fabrication of optical devices, and biomedical applications.^31–33^ Doping terbium with GQDs facilitates energy transfer between metal ions and GQDs, resulting in enhanced photoluminescence. Further, the carbon structure in GQD acts as an antenna, facilitating the transfer of absorbed energy to the metal ions (dopant), resulting in increased fluorescence sensitivity and sharper emission bands.^27^

Over a decade, very few have reported on hybrid RE-doped GQDs. For instance, Pei et al. have demonstrated Europium-functionalized GQDs for detecting cobalt ions in water and their applications in white light emission.^34^ Latif et al. reported the deferasirox sensing using Tb-doped carbon dots for ratiometric and colorimetric sensing.^30^ In addition, N-GQD/Tb- Phen, a dual-emission ratiometric fluorescent probe for metformin detection, was studied by Gazizadeh et al.^35^ L Jiang et al. have designed blue fluorescent carbon dots synthesized by the hydrothermal method with a detection limit of 42.4 and 130.15 nM via fluorescence and UV-absorption methods.^36^ In this study, citric acid and urea were employed as carbon and nitrogen precursors for the synthesis of Tb-GQDs toward the selective and sensitive detection of tartrazine. In contrast, our previous research utilized citric acid and diethylenetriamine for the preparation of Tb-GQDs aimed at the selective detection of melamine, due to the presence of amine-rich functional groups.^37^ The choice of precursors plays a significant role in influencing surface functional groups, electronic structure, and thereby changes in interaction behavior with target analytes. In general, the choice and composition of precursors significantly influence surface functional groups and electronic structure, thereby altering interaction behavior with target analytes. Specifically, variations in precursor composition can significantly alter the surface chemistry and functional groups of the resultant Tb-GQDs, impacting their analyte-specific sensing performance. Hence, understanding precursor-dependent surface characteristics is important for enhancing the sensing performance of rare-earth-doped GQDs in food safety applications. Moreover, reports on hybrid rare-earth-doped graphene quantum dots (RE-GQDs) remain scarce, and no previous studies have described the use of Tb-GQDs for tartrazine sensing in recent decades. In this study, Tb-GQDs were synthesized and evaluated as fluorescent probes for tartrazine detection in food samples, including noodles, flavored drinks, and carbonated beverages. GQDs were prepared via a one-pot carbonization process using urea and citric acid as nitrogen and carbon sources, respectively. Subsequently, Terbium was doped in GQD’s surface via active coordination sites in GQD. Comprehensive characterization of the synthesized Tb-GQDs was conducted using X-ray diffraction (XRD), high-resolution transmission electron microscopy (HR-TEM), Fourier transform infrared spectroscopy (FTIR), UV–visible spectrophotometry, and photoluminescence spectroscopy. The resulting Tb-GQDs demonstrated high selectivity and sensitivity as fluorescent probes for tartrazine detection in various real-time food samples.

## 2 Experimental Section

### 2.1 Materials and Methods

The Supporting Information provides the details of all the chemicals and reagents used in the present study.

### 2.2 Synthesis of Graphene Quantum Dots

Graphene quantum dots (GQDs) were synthesized using a one-pot carbonization method as shown in Scheme 1.^38^ A 1:1 mixture of citric acid and urea was added and heated at 200 °C in a heating crucible for 30 min. While heating the mixture, the color changed from colorless to a brownish liquid. The resulting mixture will be purified by filtering using a 0.22 μm syringe filter, and the filtrate will be stored at 4 °C in a refrigerator for further use.

**Scheme 1:**
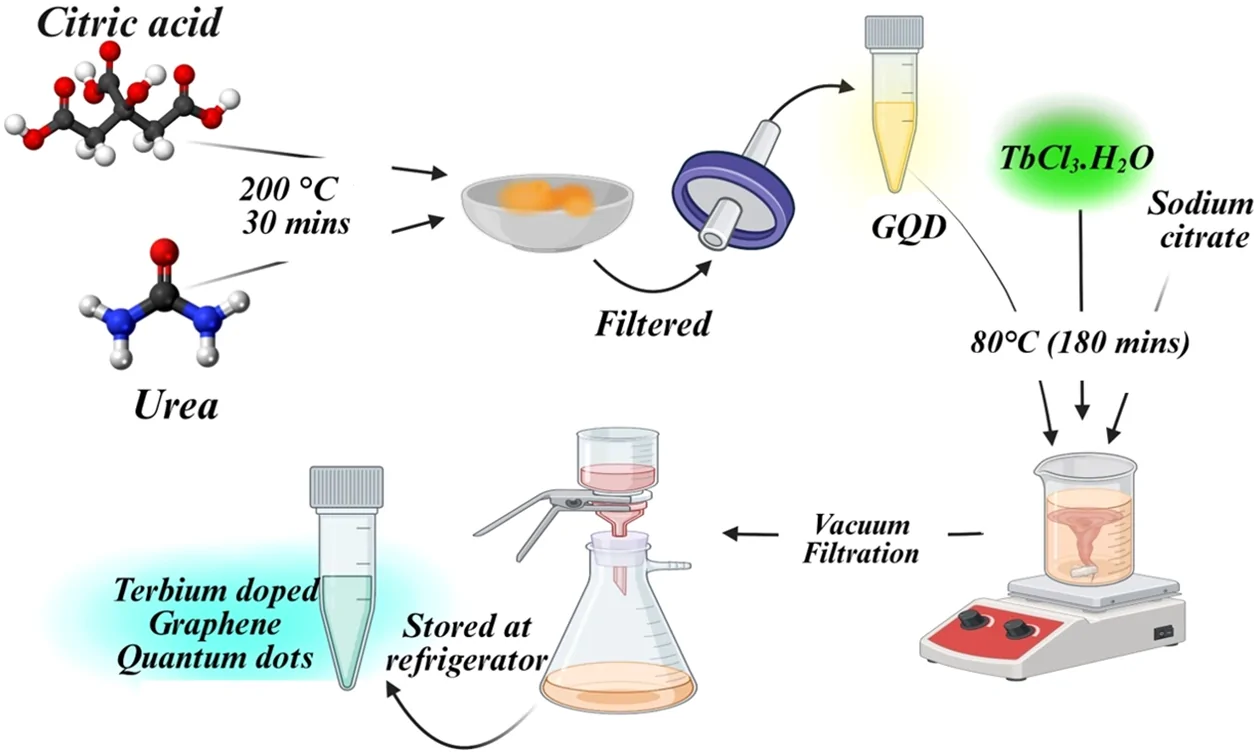
Schematic Diagram of the Synthetic Procedure of Graphene Quantum Dots and Formation of Terbium-Doped Graphene Quantum Dots

### 2.3 Formation of Terbium-Doped GQDs

Terbium was successfully doped onto GQD’s surface, as follows from our previous work.^37^ A known amount of GQD (2 mL) was diluted to 10 mL using deionized water, and the mixture was stirred magnetically at 80 °C for 180 min. After this, 0.2 mmol % of TbCl_3_·6H_2_O was added to the aforementioned solution to form terbium-doped GQDs. The pH of the homogeneous mixture was adjusted to 7.2 to 7.4 by adding 1% NaOH solution. To avoid coagulation, 2 mg of sodium citrate was added and allowed to stir for 30 min at room temperature. The final solution was stored in a refrigerator for further use.^39^

### 2.4 Quantum Yield Calculation

The quantum yield of the as-prepared GQD and Tb-GQDs was calculated by considering quinine sulfate as standard reference. In brief, the samples were dispersed in deionized water, and standard quinine sulfate was dissolved in 0.1 N sulfuric acid (Φ = 0.54%). Finally, the optical densities (O.D) of GQD, Tb-GQD, and standard quinine sulfate were recorded and maintained at or below 0.1.^40^ Both OD and fluorescence intensities were measured at a similar wavelength of 310 nm.

The quantum yield of the GQD and Tb-GQDs was calculated using the following formula:

1 $$\phi_{S}=\phi_{R}\times \frac{I_{x}}{I_{R}}\times \frac{A_{R}}{A_{x}}\times \frac{{\eta_{x}}^{2}}{{\eta_{R}}^{2}}$$

where the subscripts “*x*” and “R” are reference and sample standards, respectively, “*I*” is the measured integrated fluorescence intensity, “η” is the refractive index of the solvent (η = 1.33 for water), “ϕ” is the quantum yield, and “*A*” is the optical density.^37^

### 2.5 Fluorescence Detection of Tartrazine

To determine the sensitivity of the as-synthesized fluorescent probe, stock concentrations of tartrazine (0.25 mg mL^–1^) and Tb-GQD (0.5 mg mL^–1^) were prepared. The Tb-GQDs were optimized under different ratios of Tb-GQD with deionized water and finalized as 0.5 mg/mL. Then, the as-prepared Tb-GQDs stock was dispersed in 20 mL of deionized water, followed by sonication for 15 min. Various concentrations of tartrazine (0.5–110 μM) were added to 500 μL of Tb-GQDs. A series of tartrazine concentrations with Tb-GQDs was prepared, making up to 3 mL, using DI water. After sonicating the samples for 10 min, fluorescence emission spectral changes were recorded upon excitation at 310 nm.

### 2.6 Detection of Tartrazine in Food Samples

Most of the food samples have added tartrazine in their list of ingredients using various labels such as INS 102, E-102, and FD&C Yellow No.5. To evaluate the presence of Tartrazine in the commercial food products, a mango-flavored drink, an energy drink, and a masala powder for noodles were purchased from our nearby departmental store in Vellore. To extract the supernatant, the purchased sample and its solvent were combined in a 1:10 ratio and centrifuged. The three food samples were further filtered using vacuum filtration, and the resultant solution was stored in a refrigerator at 4 °C.

### 2.7 Instrumentation

The physicochemical characteristics of Tb-GQD were analyzed using various methods, including X-ray diffraction of synthesized GQD and Tb-GQD, which was measured using a Bruker D8 Advance with Cu Kα radiation. The Tb-GQD morphology was examined by HR-TEM using an FEI Tecnai G2 20 S-TWIN (200 kV). The average particle size of Tb-GQD was determined using ImageJ. A Fourier transform infrared spectrophotometer was used to analyze surface functional groups, purchased from Thermo Fisher Scientific Pvt Ltd. (Thermo Nicolet iS50 with built-in ATR). The Heber multilight photoreactor was used to analyze the photostability of the material under continuous irradiation of 254 nm. The absorption spectra were recorded using a UV–vis-NIR spectrophotometer (Agilent Cary 5000). ULVAC-PHI Versa Probe 4 was used to record the X-ray photoelectron spectroscopy. FLS 1000-SS-s from Edinburgh Instruments was used to obtain the photoluminescence spectra and lifetime measurements. The schematic illustrations were created using BioRender Software.

## 3 Results and Discussion

### 3.1 Structural Characteristics

The crystalline nature of both GQD and Tb-GQD was analyzed using X-ray diffraction spectroscopy, as shown in Figure 1a. The bare GQD exhibits a broad diffraction peak centered at 2θ ≈ 27°, corresponding to the (002) lattice plane. It confirms the presence of smaller graphitic carbon, consistent with previous reports.^41^ Upon addition of terbium onto the GQDs, there is an existence of a broad diffraction peak around ∼25–27°, and a new sharp diffraction peak at ≈31.7° and 45° indicates the presence of terbium onto the GQD surface. The presence of a broad (002) band along with distinct Tb-related reflections indicates that Tb ions are doped onto the GQD surface without affecting the graphitic lattice (Figure 1a). Both GQD and Tb-GQD display similar XRD patterns that match with JCPDS No.00–048–1206 and 01–071–3930, further supporting the formation of Tb-GQD.^42^

**Figure 1:**
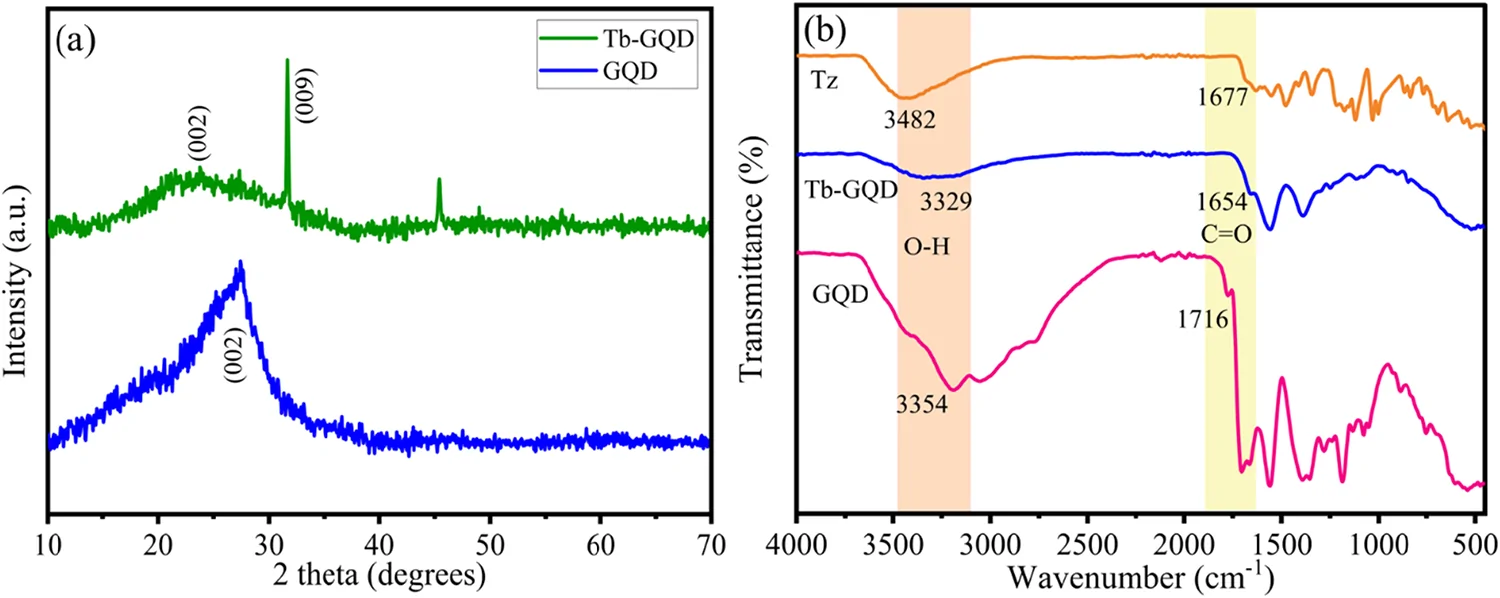
(a) XRD pattern of GQD and Tb-GQDs. (b) FTIR spectrum of GQD, Tb-GQD, and tartrazine (Tz).

From Figure 1b, the surface functional groups of GQD, Tb-GQD, and tartrazine were analyzed using FTIR spectra. From the figure, it is observed that GQD and Tb-GQD exhibit a broad O–H stretching band centered around ∼3425 and ∼3385 cm^–1^, respectively, corresponding to surface hydroxyl and carboxylic groups. Alongside, the peaks around ∼1716 and ∼1690 cm^–1^, present in both GQD and Tb-GQD, are attributed to the C = O stretching band.^43^ A noticeable red shift was observed, indicating the presence of terbium ions on the GQD surface. From Figure 1b, the peak (red line) at 1119 cm^–1^ corresponds to C–N stretching, 1033.8 cm^–1^ is linked to C–O, and the S = O group confirms the presence of tartrazine (Tz)^44^

Alongside, the HR-TEM images of GQD and Tb-GQD, revealing a quasi-spherical shape and the distinctive properties of GQDs synthesized using bottom-up routes, are shown in Figure 2. Figure 2a–c shows the HR-TEM image and histogram of GQD, and Figure 2d–f represents the HR-TEM image and the histogram of Tb-GQD. The histogram in Figure 2c,f shows average particle sizes of GQD and Tb-GQD of 3.46 ± 0.22 and 3.9 ± 0.62 nm, respectively. An interplanar spacing of 0.28 nm corresponds to the in-plane graphitic lattice of sp^2^ carbon domains in Tb-GQDs. In addition, the EDS spectrum of Tb-GQD was analyzed and confirmed the presence of Terbium in GQDs, as shown in Figure S6.

**Figure 2:**
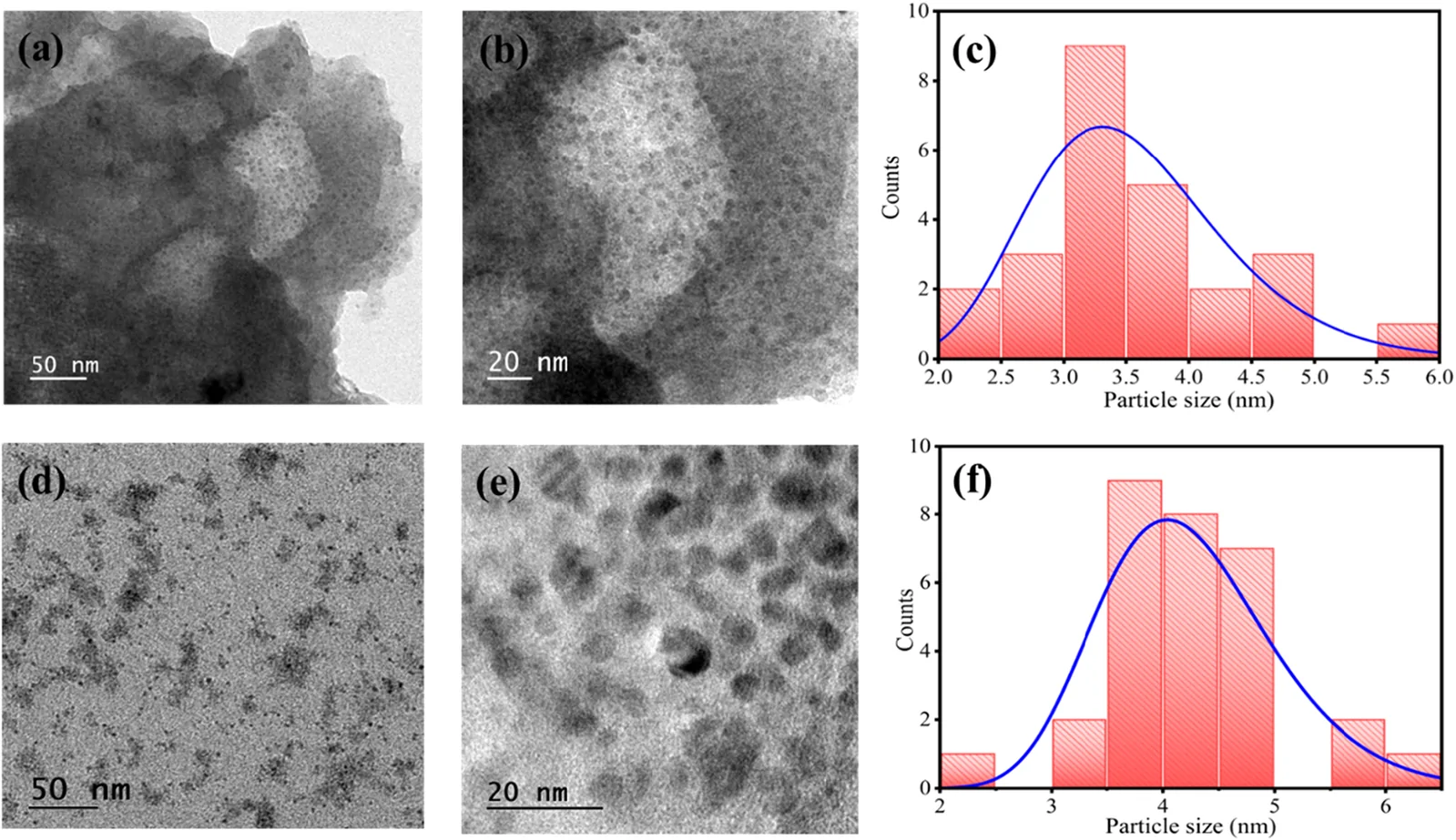
(a, b) HR-TEM images of GQD 50 and 20 nm, (c) particle size histogram of GQD, (d, e) HR-TEM images of Tb-GQD at 50 and 20 nm, and (f) particle size histogram of Tb-GQD.

XPS was used to investigate the chemical composition of the prepared Tb-GQDs. The XPS survey spectra (Figure 3a) of Tb-GQD show the corresponding peaks for the elements carbon (C 1s), oxygen (O 1s), nitrogen (N 1s), and terbium (Tb 4d), indicating the successful formation of Tb-GQDs. The elemental composition of Tb-GQDs has been identified. The C 1s spectra show peaks centered around 283.4, 284.7, and 286.9 eV, which are attributed to the graphitic C–C/C = C, C–N, and C = O/C–N, respectively.^45–48^ A peak at 283.2 eV was observed with a slight shift from the typical carbon position, which may be due to delocalization of electrons around sp^2^ carbon.^47^ In the N 1s spectrum, two distinct peaks were found around 398.8 and 400.2 eV, attributed to the pyridine N/amine and C–N, respectively. The O 1s spectra show peaks around 530.1 and 532.2 eV corresponding to metal-O (Tb–O) and C–O, as shown in Figure 3c. In Figure 3d, peaks around 148.5 and 152.5 eV confirm the presence of terbium ions, as shown in Figure 3d.^42^ The obtained results confirmed the surface functional groups and presence of terbium in the GQDs surface.

**Figure 3:**
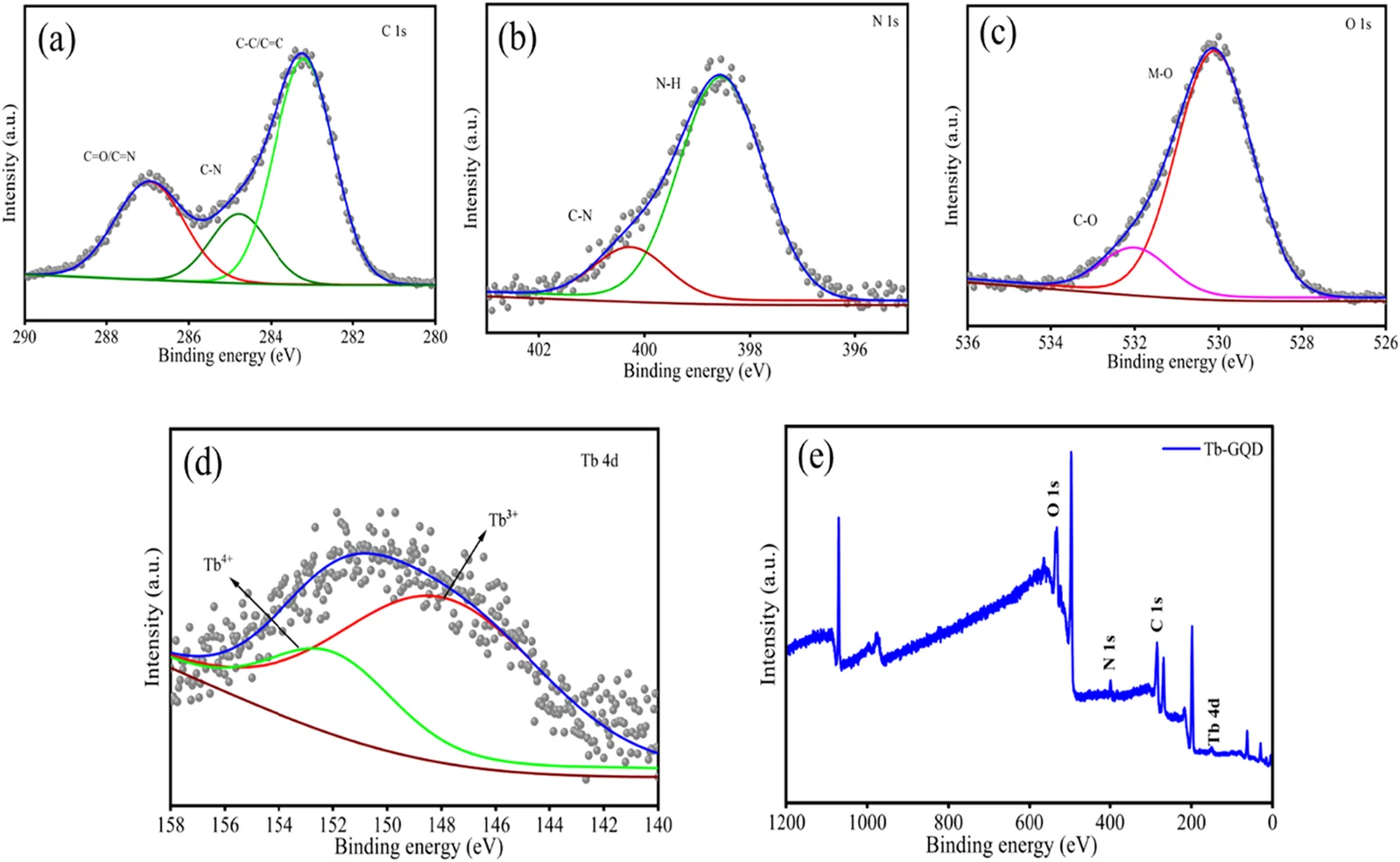
XPS spectrum of Tb-GQDs: (a) C 1s spectrum, (b) N 1s spectrum, (c) O 1s spectrum, and (d) Tb 4d spectrum. (e) Survey spectrum of Tb-GQD.

### 3.2 Optical Characteristics

The optical properties of GQDs and Tb-GQDs were recorded using a UV–visible absorption spectrophotometer and a photoluminescence emission spectroscope. Figure 4a shows pristine GQDs exhibiting two distinct peaks at 230 and 337 nm. These peaks correspond to the π–π* transition of aromatic C = C bonds in graphene and the n–π* transition attributed to the C = O bond in the carbonyl group, respectively.^49^ In addition, the absorption maximum of Tb-GQDs at 337 nm exhibits a broad spectrum with a slight increase in absorbance and an increase in the full width at half-maximum (fwhm). This clearly indicates the coordination of Tb^3+^ ions with oxygenated surface groups.

**Figure 4:**
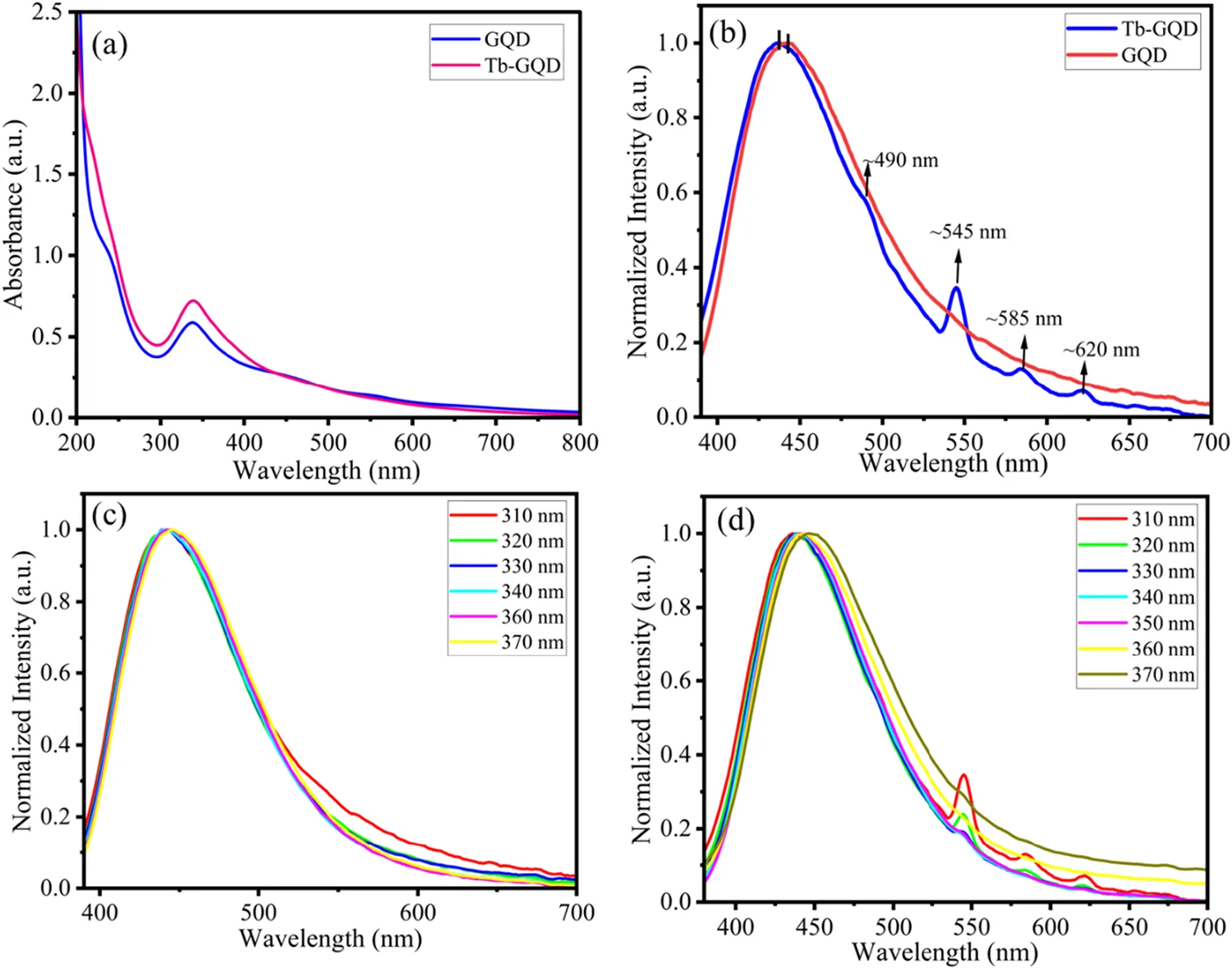
(a) Absorbance spectra of GQD and Tb-GQD, (b) photoluminescence emission spectra of GQD and Tb-GQD, (c) emission map of GQD, and (d) emission map of Tb-GQD.

Figure 4b shows the photoluminescence emission spectra of GQD and Tb-GQD. Upon excitation at 310 nm, a broad emission peak centered at 442 nm was observed in bare GQD, whereas Tb-GQD shows the emission peaks at 490, 545, 585, and 620 nm, respectively,^50^ which are attributed to ^5^D_4_ → ^7^F_6_, ^5^D_4_ → ^7^F_5_, ^5^D_4_ → ^7^F_4_, and ^5^D_4_ → ^7^F_3_ transitions of Tb^3+^ ions along with the GQD peak at 440 nm.^51^ The fluorescence quantum yield of GQD and Tb-GQD is calculated as 17.45 and 31.2%, respectively, using quinine sulfate as a standard reference.

In addition, the excitation-dependent emission from both GQD and Tb-GQD is shown in Figure 4c,d. The emission spectra were observed upon excitation at wavelengths from 310 to 370 nm. Upon exciting the Tb-GQDs at different wavelengths, the emission spectrum shows a slight red shift and increasing emission intensity, clearly demonstrating the distinctive feature of the quantum-confined structure (normalized emission spectra are shown in Figure 4d). The red shift observed as the excitation wavelength increases indicates a distribution of terbium ions on the surfaces and within the emissive sites of the GQDs. In particular, when exciting the Tb-GQDs at 310 nm, the terbium peak maxima at 543, 585, and 620 nm are observed, and this λ_ex_ is used for tartrazine detection studies throughout this work. In contrast, the bare GQDs showed no change in the emission wavelength, although the excitation wavelength increased (Figure 4c). This study clearly confirms the energy transfer from GQD to Tb^3+^ centers and modifies the optical characteristics of GQDs.

### 3.3 Stability Analysis of Tb-GQD

The photostability of bare GQD and Tb-GQD was evaluated under continuous irradiation at 254 nm, a high-energy UV exposure, as shown in Figure 5a. Bare GQDs exhibit a consistent and noticeable drop in fluorescence intensity from 1.0 to 0.62 across the entire emission region, observed 38% loss over 90 min. In contrast, Tb-GQD shows a fluorescence intensity drop from 1.0 to 0.76, observed 24% loss over 90 min. The results demonstrate that Tb-GQD shows better resistance to photobleaching than GQDs. In Tb-GQDs, the GQD-centered emission (445 nm) gradually decreases in intensity as the irradiation period increases, suggesting that it is susceptible to photobleaching under high-energy UV exposure. On the other hand, Tb^3+^ emission (545 nm) exhibits minimal intensity reduction, indicating an improved resistance to photodegradation (Figure 5b). This discrepancy results from the 4f electronic transitions in Tb^3+^ being shielded, rendering them less vulnerable to environmental influences. The stability of Tb-GQD is due to the efficient energy transfer from the GQD matrix to Tb^3+^ ions. It is confirmed that incorporating terbium significantly increases the photostability of the GQDs, making them more suitable for sensing applications.

**Figure 5:**
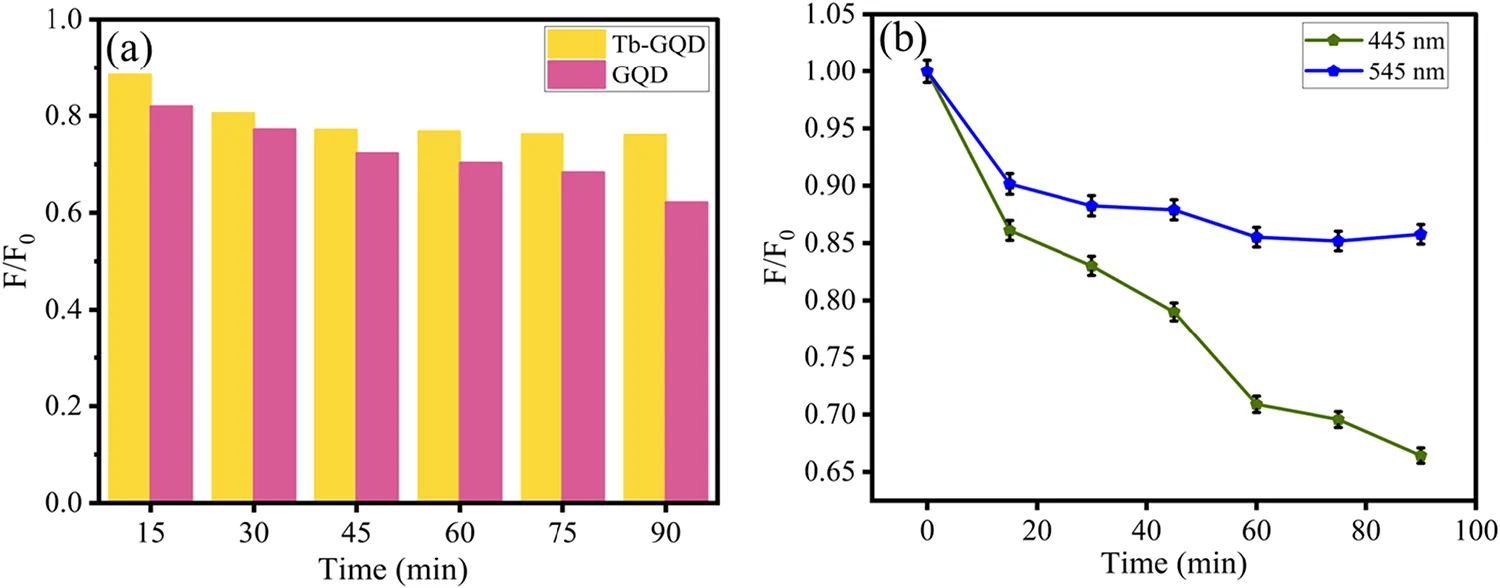
(a) Comparative photostability analysis of GQD and Tb-GQD under continuous irradiation of 254 nm. (b) Time-dependent photostability of Tb-GQD under continuous UV irradiation, observed at GQD emission (445 nm) and Tb^3+^ emission (545 nm) wavelengths.

Moreover, the temperature stability and interday and intraday stability were examined to observe the Tb-GQDs’ stability and repeatability. Figure S7 shows the temperature analysis of Tb-GQDs, and it is observed that there is no significant change in fluorescence emission intensity by altering the temperature up to 60 °C. Table S1 shows the interday and intraday analysis of Tb-GQD with tartrazine, which shows a good recovery range of 93.7–98.2%, ensuring the repeatability of Tb-GQD.

### 3.4 Interaction Studies

Tb-GQD with varying concentrations of tartrazine (0.5–110 μM) was recorded using a UV–visible spectrophotometer and photoluminescence spectroscopy. Figure 6a shows the absorbance spectra of Tb-GQDs with increasing concentration of tartrazine. The absorption maxima of Tb-GQD were recorded at 335 nm, and those of tartrazine were around 270 and 425 nm. As the concentration of tartrazine increases, the absorption maxima show a hyperchromic and bathochromic shift toward longer wavelengths. This bathochromic shift may be due to the ground-state complex formation between Tb-GQDs and tartrazine moieties. The absorption spectra of Tb-GQD, tartrazine, and Tb-GQD-tartrazine are shown in Figure 6(b, inset), respectively. In addition, the photoluminescence emission spectra of Tb-GQDs at increasing concentrations of tartrazine are shown in Figure 6b. From Figure 6b, the photoluminescence emission spectra of Tb-GQDs show a broad peak at 435 nm in the GQD region, along with multiple sharp peaks at ∼490, ∼545, ∼585, and ∼620 nm, indicating efficient energy transfer from GQD to Tb^3+^ ions. Upon addition of tartrazine, the maximum fluorescence intensity of Tb-GQD gradually decreases and shifts to longer wavelengths, further confirming its efficient quenching. From Figure 6b, the linear plot between (*F*_o_–*F*)/*F*_o_and concentrations of tartrazine was used to calculate the detection limit, and the correlation coefficient (*R* ^2^) was observed to be 0.9910, as shown in Figure S1.

2 LOD=3σ/m

where *m* represents the slope of the linear plot and σ denotes the standard deviation of Tb-GQD. The detection limit is calculated as ∼6.7 nM using the above formula.

**Figure 6:**
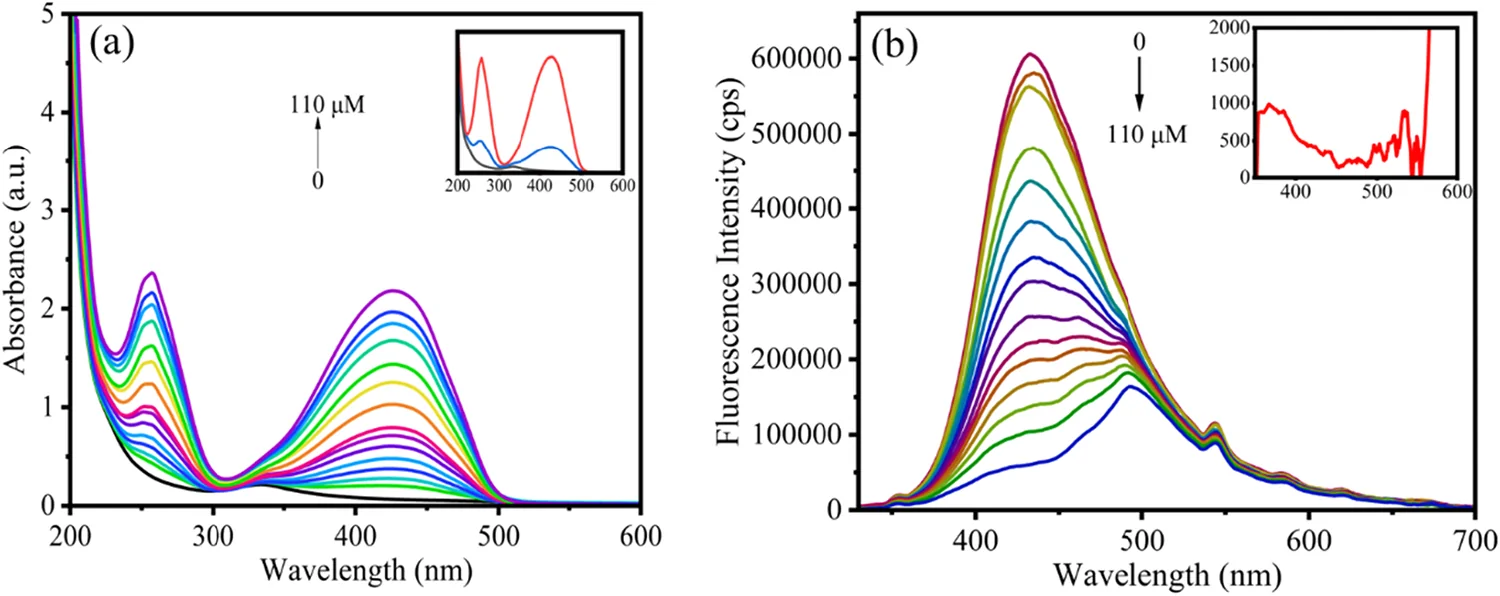
Tb-GQDs with different concentrations of tartrazine: (a) UV–visible interaction spectra and (b) fluorescence spectra.

### 3.5 Selective Detection of Tartrazine

To evaluate the selectivity of Tb-GQD with tartrazine, a synthesized Tb-GQDs were added to various commonly found food additives, biomolecules and metal ions such as (A) Tb-GQD, (B) citric acid, (C) Fe^2+^, (D) Co^2+^, (E) butylated hydroxyanisole, (F) glycine, (G) *tert*-butylhydroquinone, (H) Allura red, (I) l-arginine, (J) Ni^2+^, (K) dopamine, (L) Sunset yellow, (M) Bisphenol A, (N) glutamic acid, (O) vanillin, (P) monosodium glutamate, (Q) Melamine, (R) Cu^2+^, (S) Cd^2+^, (T) Ca^2+^, and (U) Tartrazine (Tz). The desired concentration (50 μM) of various molecules was added to a fixed concentration of Tb-GQDs (500 μL), and the photoluminescence emission was recorded under the excitation wavelength of 310 nm. As shown in Figure 7, the emission intensity of Tb-GQD shows negligible variation in the presence of various analytes. In contrast, the presence of tartrazine results in a significant decrease in fluorescence intensity compared to other species. This shows that Tb-GQD is highly selective toward the sensing of tartrazine moieties.

**Figure 7:**
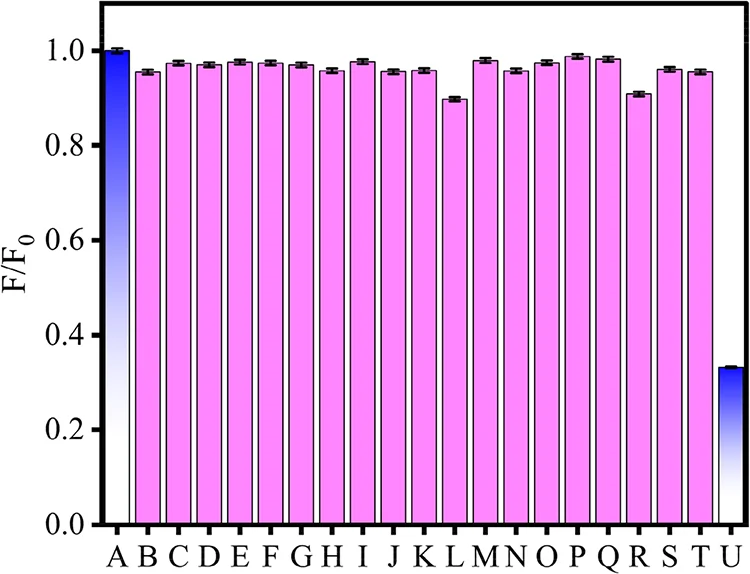
Selectivity studies of Tb-GQD toward tartrazine.

### 3.6 Fluorescence Lifetime Analysis

Fluorescence lifetime spectroscopy (FLS) helps determine the fluorescence decay time of nanomaterials and the time spent in the excited state. The fluorescence lifetime of Tb-GQD and Tb-GQD-tartrazine was recorded using a fluorescence steady-state spectrometer, and the decay spectra are shown in Figure 8. The Tb-GQDs exhibit triexponential decay due to multiple emissive sites attributed to terbium ions on the GQDs. Table 1 shows the lifetime measurements of Tb-GQD and Tb-GQD-tartrazine. It is observed that there is no significant variation in the average fluorescence lifetimes of Tb-GQD and Tb-GQD-tartrazine, which were found to be ∼0.46 μs and ∼0.44 μs when adding tartrazine.

**Table 1:** Fluorescence Lifetime Decay Parameters of Tb-GQD and Tb-GQD with Tartrazine

sample	τ1 (ns)	τ2 (ns)	τ3 (ns)	α1	α2	α3	average lifetime (μs) $\tau_{0}=\sum_{i=1}^{2}\frac{A_{i}\tau_{i}^{2}}{A_{i}\tau_{i}}$	χ^2^
Tb-GQD	259	109	10220	0.860	0.130	0.009	0.46	1.2
Tb-GQD + tartrazine	212	1014	10850	0.821	0.169	0.009	0.44	1.1

**Figure 8:**
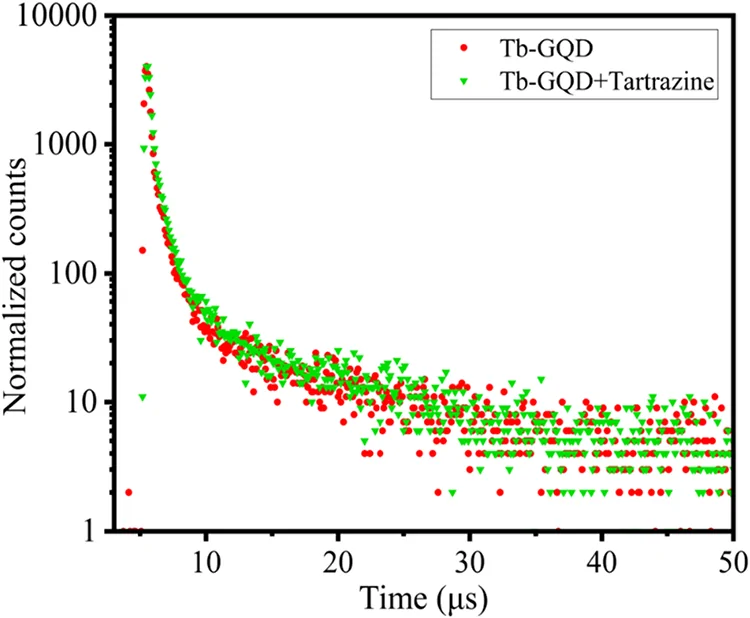
Fluorescence lifetime spectra of Tb-GQD and Tb-GQD with tartrazine.

### 3.7 Sensing Mechanism of Tartrazine

Generally, fluorescence quenching can result from various phenomena such as energy transfer, static quenching, and dynamic quenching. The plausible quenching mechanism was investigated using UV–visible and photoluminescence spectroscopy, fluorescence lifetime measurements, and Stern–Volmer studies.^52^ Figure S2 shows the overlapping of the absorbance of tartrazine and the excitation and emission of Tb-GQD, suggesting that quenching occurred due to the inner filter effect (IFE) or Forster resonance energy transfer (FRET). As IFE is based on distance-independent quenching, FRET is due to the fluorescent probe-quencher distance. Here, the overlap is between the absorbance and excitation of Tb-GQD and the unchanged lifetime of Tb-GQD and Tb-GQD with tartrazine, which shows the chances of IFE.^53^

Further, a Stern–Volmer investigation is carried out to understand the quenching of Tb-GQD upon the addition of various concentrations of tartrazine (0.5–110 μM).

Stern–Volmer (S–V) constant was calculated using the following equation:

3 $$\frac{F_{0}}{F}=1+\mathrm{Ksv}\left[Q\right]$$

where *F*_o_ and *F* are the fluorescence intensities of Tb-GQD alone and different ratios of Tb-GQD with tartrazine, respectively, [*Q*] denotes the concentration of tartrazine, and Ksv refers to the Stern–Volmer constant. Figure S3 shows the upward curvature while plotting between F0/F and various concentrations of tartrazine, and it indicates that quenching can happen due to multiple pathways. The average lifetime values of Tb-GQD and Tb-GQD with tartrazine are 0.46 and 0.44 μs, respectively, suggesting that the fluorescence quenching process does not happen due to collisional interactions or dynamic quenching. A linear plot between Fo/F vs lower concentration of tartrazine was used to obtain the Stern–Volmer constant (*Ksv*, in L mol^–1^) and correlation coefficient (*R* ^2^ = 0.9956) (using eq 3), as shown in Figure S4. At lower concentrations of tartrazine, the Stern–Volmer plot is obtained linearly with an intercept of 1, indicating that the lower concentration is predominantly static in nature

From the modified Stern–Volmer equation,

4 $$\log\frac{\left(F_{0}-F\right)}{F}=\log K_{a}+n\;\log\left[\mathrm{conc}\right]$$

Figure S5 shows the plot between log [conc] and log [*F*_0_–*F*/*F*]. From the linear plot, the binding constant *K*_a_ = 2.8 × 10^4^ M^–1^ and the number of binding sites *n* = 1.18 are calculated. These results support the formation of a ground-state complex.^50,54^ Further, the UV–visible interaction studies of Tb-GQD with tartrazine show a bathochromic shift due to the formation of a nonfluorescent ground-state complex. Hence, the fluorescence lifetime and Stern–Volmer studies suggest that it is predominantly static with IFE contribution. Table 3 presents comparative reports on previously reported fluorescence sensing of tartrazine.

### 3.8 Detection of Tartrazine in Real Samples

The practical implementation of the proposed analytical method was evaluated using a standard addition approach for commercially available flavored and carbonated soft drinks, as well as in noodle powder. Spectroscopic analysis was performed on both pretreated samples and tartrazine-spiked pretreated food samples. The pretreated unspiked sample may contain negligible amounts, or the presence may be below the limit of detection. The pretreated food samples were added to a known concentration of tartrazine that had been previously mixed with Tb-GQDs. Table 2 shows that recovery ranged from 99.4 to 101.5%, and the relative standard deviation (RSD) was below 1.2% for most samples. The results demonstrate the effectiveness, reliability, and quantified reproducibility of Tb-GQDs as a sensing platform for detecting tartrazine in various food samples with good selectivity and sensitivity. The overall observation suggests that this method is reliable for sensing tartrazine in complex food matrixes.

**Table 2:** Detection of Tartrazine in Various Food Samples

real samples	concentration added (μM)	concentration obtained (μM)	recovery (%)	RSD (%) *n* = 3
pretreated sample	0	0		
carbonated drinks	10	9.94 ± 0.02	99.4	0.19
	20	20.17 ± 0.04	100.8	0.2
	25	24.88 ± 0.1	99.5	0.42
flavored drink	10	9.74 ± 0.19	97.4	1.97
	20	20.3 ± 0.23	101.5	1.13
	25	24.43 ± 0.09	97.7	0.37
noodles masala powder	10	10.08 ± 0.02	100.8	0.2
	20	19.83 ± 0.19	99.1	0.89
	25	24.65 ± 0.11	98.6	0.44

**Table 3:** Comparison of LOD with Previously Reported Tartrazine Detection

material	detection technique	linear range (μM)	limit of detection (LOD)	references
fluorescent CDs (m-phenylenediamine)	fluorescence (dynamic + IFE)	0.01–25.0	12.4 nM	^55^
sulfur quantum dots (SQDs)	fluorescence (IFE)	0.10–20	39 nM	^1^
carbon dots (Aloe)	static quenching	0.25–32.50		^56^
carbon dots (Gandha Prasarini leaves)	fluorescence (IFE)		0.18 μM	^57^
B–O–CDs	IFE	0.2–60	64 nM	^58^
dual-emission CDs (DE-CDs)	IFE	0–200	0.10 μM	^59^
Tb-GQDs	fluorescence (static + IFE)	0.5–110	6.7 nM	this work

## 4 Conclusion

This study demonstrates the performance of Tb-GQDs as an ultrasensitive fluorescent platform for tartrazine detection in various food samples. The precursors used for the synthesis were quite cost-effective and easily available. In brief, the as-synthesized Tb-GQDs exhibit uniform nanoscale morphology with an average particle size of approximately 3.9 ± 0.62 nm, which is potentially suitable for biomedical applications. Further, FTIR analysis confirms the effective surface functionalization and interactions between Tb-GQDs and tartrazine, thereby increasing the selectivity of the sensing material. The applicability of the proposed method was validated using real food matrices, including flavored drinks, carbonated beverages, and noodle masala powder, yielding satisfactory recoveries of 97.4 to 101.5% and relative standard deviations below 1.2%, indicating good accuracy and precision. Overall, the developed Tb-GQD-based fluorescent probe provides a rapid, reproducible, and selective approach for routine tartrazine analysis, addressing key analytical limitations associated with the detection of synthetic food colorants in food safety monitoring. Future studies will focus on the design and fabrication of a cost-effective, highly compatible Tb-GQD-based fluorescent sensor for tartrazine detection.

## Supplementary Material



## Notes

Materials and methods, linear plot of (*F*_o_–*F*)/*F*_o_ vs concentration of tartrazine (Figure S1), absorbance spectrum of Tb-GQD and tartrazine, the emission and excitation spectrum of Tb-GQD (Figure S2), Stern–Volmer plots (Figures S3 and S4), modified Stern–Volmer plot (Figure S5), EDS spectrum of Tb-GQD (Figure S6), and stability analysis (temperature, pH, intraday and interday) of Tb-GQD (Figures S7 and S8, Table S1) (PDF)

## Abbreviation

GQD: graphene quantum dots
Tb-GQD: terbium-doped graphene quantum dots
RE: rare earth
XRD: X-ray diffraction
UV–Vis: ultraviolet–visible
HRTEM: high-resolution transmission electron microscopy
Tz: tartrazine
FSSAI: food safety and standards authority of India
HPLC: high-performance liquid chromatography
TLC: thin-layer chromatography
LC-MS: liquid chromatography–mass spectrometry
PL: photoluminescence
REE: rare earth elements
pH: power of hydrogen
FTIR: Fourier transform infrared
UV–vis-NIR: ultraviolet–visible-near-infrared
LOD: limit of detection
FLS: fluorescence lifetime spectroscopy
IFE: inner filter effect
FRET: fluorescence resonance energy transfer
RSD: relative standard deviation
XPS: X-ray photoelectron spectroscopy

## References

[1] Peng X., Wang Y., Wang Q., Tang J., Zhang M., Yang X. (2022). Selective and Sensitive Detection of Tartrazine in Beverages by Sulfur
Quantum Dots with High Fluorescence Quantum Yield. Spectrochim. Acta, Part A.

[2] Zoughi S., Faridbod F., Amiri A., Ganjali M. R. (2021). Detection of Tartrazine
in Fake Saffron Containing Products by a Sensitive Optical Nanosensor. Food Chem..

[3] Chaudhari S. S., Patil P. O., Bari S. B., Khan Z. G. (2024). A Comprehensive
Exploration of Tartrazine Detection in Food Products: Leveraging Fluorescence
Nanomaterials and Electrochemical Sensors: Recent Progress and Future
Trends. Food Chem..

[4] Thulasi S., Kathiravan A., Asha Jhonsi M. (2020). Fluorescent Carbon Dots Derived from
Vehicle Exhaust Soot and Sensing of Tartrazine in Soft Drinks. ACS Omega.

[5] Zingue S., Mindang E. L. N., Awounfack F. C., Kalgonbe A. Y., Kada M. M., Njamen D., Ndinteh D. T. (2021). Oral Administration
of Tartrazine
(E102) Accelerates the Incidence and the Development of 7,12-Dimethylbenz­(a)
Anthracene (DMBA)-Induced Breast Cancer in Rats. BMC Complementary Med. Ther..

[6] Liu H., Li Z., Zhang W., Liu Y., Pan R., Huang G. (2022). Facile Synthesis
of Tomato-Based Carbon Nanodots and Its Utilization in Sensitive Detection
of Tartrazine. J. Indian Chem. Soc..

[7] Amchova P., Kotolova H., Ruda-Kucerova J. (2015). Health Safety
Issues of Synthetic
Food Colorants. Regul. Toxicol. Pharmacol..

[8] Deng K., Li C., Li X., Huang H. (2016). Simultaneous Detection of Sunset
Yellow and Tartrazine Using the Nanohybrid of Gold Nanorods Decorated
Graphene Oxide. J. Electroanal. Chem..

[9] Li C., Xia S., Qian X., Zheng M., Deng K. (2025). Fe3O4/CoFe Nanoparticles
Decorated 3D Hierarchical Nitrogen-Doped Carbon Nanosheets for Synchronous
Measurement of Sunset Yellow and Tartrazine. Ionics.

[10] Zhang H., Wang J., Wu W., Han C., Li M. (2025). Graphene Oxide
Supported MOFs-Nanofiber Carbon Aerogel/SPCE for Simultaneous Detection
of Cd2+ and Pb2+ in Seafood. Food Chem..

[11] Uppada, H. ; Nasu, S. ; Saripilli, P. ; Sushmabee, S. Analytical insights into tartrazine detection in food and beverages. 2024.

[12] Palma P., Godoy M., Calderón R. (2024). Simultaneous
Determination of 11
Water-Soluble Synthetic Colorants in Foods Consumed in Chile by High-Performance
Liquid Chromatography with Diode Array Detection. Food Chem..

[13] HURST W. J., McKIM J. M., MARTIN R. A. (1981). Determination of Tartrazine in Food
Products by HPLC. J. Food Sci..

[14] Guo Y., Huang Q., Xu F., Luo Z., Wei Y., Chen Z., Zeng Z., Zhang H., Shi H. (2025). A Graphene
Quantum Dots Based Dual-Modal Fluorometric and Visualized Detection
of Copper Ions. Spectrochim. Acta, Part A.

[15] Geng Z. (2025). A Highly Sensitive
Fluorescent Detection Method Utilizing B and S Co-Doped Graphene Quantum
Dots for Ibuprofen Analysis. Alexandria Eng.
J..

[16] Zhang Z., Fan Z. (2021). Application of Cerium–Nitrogen
Co-Doped Carbon Quantum Dots
to the Detection of Tetracyclines Residues and Bioimaging. Microchem. J..

[17] Bogireddy N. K. R., Lara J., Fragoso L. R., Agarwal V. (2020). One-Step Hydrothermal
Preparation of Highly Stable N Doped Oxidized Carbon Dots for Toxic
Organic Pollutants Sensing and Bioimaging. Chem.
Eng. J..

[18] Nan H. R., Liu Y. H., Gong W. J., Peng H. B., Wang Y. Q., Zhang Z., Bin, Cao X. H. (2022). An Inner-Filter-Effect
Based Ratiometric
Fluorescent Sensor for the Detection of Uranyl Ions in Real Samples. Anal. Methods.

[19] Pierrat P., Gaumet J. J. (2020). Graphene Quantum Dots: Emerging Organic Materials with
Remarkable and Tunable Luminescence Features. Tetrahedron Lett..

[20] Zhang H., Wang J., Ji X., Bao Y., Han C., Sun G. (2024). Nitrogen and Sulfur Co-Doped Graphene-Quantum-Dot-Based
Fluorescent
Sensor for Rapid Visual Detection of Water Content in Organic Solvents. Molecules.

[21] Das R., Paria A., Giri P. K. (2025). Machine Learning Framework for Selective
and Sensitive Metal Ion Sensing with Nitrogen-Doped Graphene Quantum
Dots Heterostructure. Carbon N. Y..

[22] Shehab M., Ebrahim S., Soliman M. (2017). Graphene Quantum
Dots Prepared from
Glucose as Optical Sensor for Glucose. J. Lumin..

[23] Zhang H., Zhang Q., Ji X., Han B., Wang J., Han C. (2025). Portable Bacterial Cellulose-Based
Fluorescent Sensor for Rapid and
Sensitive Detection of Copper in Food and Environmental Samples. Molecules.

[24] Hasan M. T., Gonzalez-Rodriguez R., Lin C. W., Campbell E., Vasireddy S., Tsedev U., Belcher A. M., Naumov A. V. (2020). Rare-Earth Metal
Ions Doped Graphene Quantum Dots for Near-IR In Vitro/In Vivo/Ex Vivo
Imaging Applications. Adv. Opt. Mater..

[25] Li M., Chen T., Gooding J. J., Liu J. (2019). Review of Carbon and
Graphene Quantum Dots for Sensing. ACS Sens..

[26] Ansari L., Hallaj S., Hallaj T., Amjadi M. (2021). Doped-Carbon Dots:
Recent Advances in Their Biosensing, Bioimaging and Therapy Applications. Colloids Surf., B.

[27] Zhang M., Zhai X., Sun M., Ma T., Huang Y., Huang B., Du Y., Yan C. (2020). When Rare
Earth Meets
Carbon Nanodots: Mechanisms, Applications and Outlook. Chem. Soc. Rev..

[28] da
Rocha J. D. G., Cechinel M. A. P., Rocha L. F., Riella H. G., Padoin N., Soares C. (2024). Exploring the Potential of Rare Earth
Doped Carbon Dots: Concepts and Applications. Chem. Eng. J. Adv..

[29] Kalyanasundaram D., Arockia Sagayaraj P.
M., Dasgupta T., Tharani G. R., Ramasamy T., Sundaramoorthy A., Nandigam N., Udayakumar K., Kumar V. S., Karthikeyan S., Chinnathambi S., Pandian G. N., Grace A. N., C Sangeetha C., Mangaiyarkarasi R. (2025). Doxorubicin-Conjugated Terbium-Doped Carbon Dots for
Site-Specific Colon Cancer Theranostics. ACS
Appl. Nano Mater..

[30] Latifi F., Amjadi M., Hallaj T., Alavi S. M. S. (2025). Tb Doped Carbon
Dots as a Platform for Fluorescence Ratiometric and Colorimetric Sensor
for Deferasirox. Sci. Rep..

[31] Mohandoss S., Priyadharshini A., Velu K. S., Napoleon A. A., Roy P., Ahmad N., Khan M. R., Palanisamy S., You S. G., Kim S. C. (2025). Rare-Earth
Metal-Doped Orange Emissive
Photoluminescent Carbon Quantum Dots for Highly Sensitive Detection
of Hg2+ Ions: Multi-Color Imaging and Real Samples. Mater. Sci. Eng. B.

[32] Wang H., Gao X., Liu J., Xiong Z., Zhao L. (2024). Facile Construction
of a Bifunctional Eu-MOF for Selective Fluorescence Sensing and Visible
Light Photocatalysis of Tetracycline. J. Alloys
Compd..

[33] Younis S. A., Bhardwaj N., Bhardwaj S. K., Kim K. H., Deep A. (2021). Rare Earth
Metal–Organic Frameworks (RE-MOFs): Synthesis, Properties,
and Biomedical Applications. Coord. Chem. Rev..

[34] Pei J., Li H., Chen F., Chen Z., Yuan X., Han Z., Chen D., Yu D., Zhang D. (2024). Lanthanide Functionalized
Carbon Quantum Dots for White Light Emission, PH Sensing, and Co (II)
Detection. ACS Appl. Mater. Interfaces.

[35] Gazizadeh M., Dehghan G., Soleymani J. (2022). A Ratiometric Fluorescent Sensor
for Detection of Metformin Based on Terbium–1,10-Phenanthroline–Nitrogen-Doped-Graphene
Quantum Dots. RSC Adv..

[36] Jiang L., Yuan L., Liu Z., Xiang Y., Song F., Meng L., Tu Y. (2022). Facile Hydrothermal
Synthesis and
Purification of Fluorescent Carbon Dots for Food Colorant Tartrazine
Detection Based on a Dual-Mode Nanosensor. Anal.
Methods.

[37] Anjila
P K F., Tharani G. R., Sundaramoorthy A., Kumar Shanmugam V., Subramani K., Chinnathambi S., Pandian G. N., Raghavan V., Grace A. N., Ganesan S., Rajendiran M. (2024). An Ultra-Sensitive
Detection of Melamine in Milk Using Rare-Earth Doped Graphene Quantum
Dots- Synthesis and Optical Spectroscopic Approach. Microchem. J..

[38] Liu C., Qi L., Zhang S., Wang X., Guo X., Liu H., Shi X. (2024). Color-Tunable
Graphene Quantum Dots via One-Step Nitrogen-Induced
Intramolecular Charge Transfer for in Vivo/Vitro Aminotransferase
Fluorescence Sensing. Sens. Actuators, B.

[39] Mondal T. K., Mondal S., Ghorai U. K., Saha S. K. (2019). White Light Emitting
Lanthanide Based Carbon Quantum Dots as Toxic Cr (VI) and PH Sensor. J. Colloid Interface Sci..

[40] Ensafi A. A., Hghighat Sefat S., Kazemifard N., Rezaei B., Moradi F. (2017). A Novel One-Step
and Green Synthesis of Highly Fluorescent Carbon Dots from Saffron
for Cell Imaging and Sensing of Prilocaine. Sens. Actuators, B.

[41] Wang J., Lu C., Chen T., Hu L., Du Y., Yao Y., Goh M. C. (2018). Simply Synthesized Nitrogen-Doped
Graphene Quantum
Dot (NGQD)-Modified Electrode for the Ultrasensitive Photoelectrochemical
Detection of Dopamine. Nanophotonics.

[42] A P. M., R M. (2026). Theranostic Rare-Earth-Doped Carbon
Dots: An Effective Nanocarrier
For Methotrexate Delivery in Colon Cancer. ACS
Omega.

[43] Zhu S., Zhang J., Qiao C., Tang S., Li Y., Yuan W., Li B., Tian L., Liu F., Hu R., Gao H., Wei H., Zhang H., Sun H., Yang B. (2011). Strongly Green-Photoluminescent
Graphene Quantum Dots for Bioimaging
Applications. Chem. Commun..

[44] Yamjala K., Nainar M. S., Ramisetti N. R. (2016). Methods
for the Analysis of Azo Dyes
Employed in Food Industry – A Review. Food Chem..

[45] Wang S., Chu X., Xiang X., Cao Y. (2020). Highly Selective Antenna Effect of
Graphene Quantum Dots (GQDs): A New Fluorescent Sensitizer for Rare
Earth Element Terbium in Aqueous Media. Talanta.

[46] Wang J., Yu B., Wang F., Wang Z., Xu M., Wu Y., Zhang H. (2025). Interpenetrating Twin ZIF-8/Carbon Nanofiber Aerogel Independent
Electrochemical Sensor for the Detection of Heavy Metal Ions in Soil
and Crops. Chem. Eng. J..

[47] Susi T., Kaukonen M., Havu P., Ljungberg M. P., Ayala P., Kauppinen E. I. (2014). Core Level
Binding Energies of Functionalized
and Defective Graphene. Beilstein J. Nanotechnol..

[48] Walter M., Mangolini F., McClimon J. B., Carpick R. W., Moseler M. (2025). Origin of
C­(1s) Binding Energy Shifts in Amorphous Carbon Materials. Phys. Rev. Mater..

[49] Anwar M., Shah S. I. A., Hussain I., Ahmad W., Shah N. S., Al-Anazi A., Khan J. A. (2026). Synthesis
of Nitrogen-Doped Graphene
Oxide for Photocatalytic Degradation of Atrazine: Kinetics and Mechanism. Diam. Relat. Mater..

[50] Mangaiyarkarasi R., Chinnathambi S., Aruna P., Ganesan S., Mangaiyarkarasi R., Chinnathambi Á.
S., Aruna Á. P., Ganesan Á. S. (2015). Synthesis of 5-Fluorouracil Conjugated
LaF3:Tb3+/PEG-COOH
Nanoparticles and Its Studies on the Interaction with Bovine Serum
Albumin: Spectroscopic Approach. J. Nanopart.
Res..

[51] Tagit O., Annio G., Hildebrandt N. (2015). Terbium to
Quantum Rod Förster
Resonance Energy Transfer for Homogeneous Bioassays with Picomolar
Detection Limits. Microchimica Acta.

[52] Liu X., Lu Z., Huang S., Chen N., Xiao X., Zhu X., Zhang R. (2025). A Practical Fluorometric and Colorimetric Dual-Mode Sensing Platform
Based on Two-Dimensional Porous Organic Nanosheets for Rapid Determination
of Trifluralin. Anal. Methods.

[53] Liu T., Zhang S., Fan X., Yang D., Wang M., Shao X., Wang S., Yue Q. (2022). Inner-Filter Effect
Induced Fluorescence Quenching of Carbon Dots for Cr­(VI) Detection
with High Sensitivity. J. Fluoresc..

[54] Barbero N., Barni E., Barolo C., Quagliotto P., Viscardi G., Napione L., Pavan S., Bussolino F. (2009). A Study of
the Interaction between Fluorescein Sodium Salt and Bovine Serum Albumin
by Steady-State Fluorescence. Dyes Pigments.

[55] Liu L., Sun H., Xiao L., Yang Z. quan., Han J., Gong X., Hu Q. (2021). Development of a Highly Sensitive Fluorescence Method for Tartrazine
Determination in Food Matrices Based on Carbon Dots. Anal. Bioanal. Chem..

[56] Xu H., Yang X., Li G., Zhao C., Liao X. (2015). Green Synthesis
of Fluorescent Carbon Dots for Selective Detection of Tartrazine in
Food Samples. J. Agric. Food Chem..

[57] Mohanta T., Behuria H. G., Sahu S. K., Jena A. K., Sahu S. (2023). Green Synthesis
of N,S-Doped Carbon Dots for Tartrazine Detection and Their Antibacterial
Activities. Analyst.

[58] Safaei A., Giyahban F., Ebrahimzadeh H. (2025). Development
of a Ratiometric Fluorescence
Sensor Based on Blue- and Orange-Emissive Carbon Dots for the Determination
of Tartrazine in Food Products. Food Chem..

[59] Chen Z., Peng Y., Liu S., Wang X., Guo Y. (2026). Synthesis
of Dual-Emission Carbon Quantum Dots for Ratiometric Fluorescence
Detection of Tartrazine. Dyes Pigm..

