# Supplementary material for: Highly Fluorescent Terbium-Doped Graphene Quantum Dots (Tb-GQD) for Rapid and Selective Detection of Tartrazine in Food Matrices
Source: ACS Omega. 2026 Aug 3;11(32):48183–92. doi: 10.1021/acsomega.6c04540 (PMC13491501; doi:10.1021/acsomega.6c04540)
Supplement: Supplementary file 1 [file ao6c04540_si_001.pdf]

Highly Fluorescent Terbium-doped Graphene quantum dots (Tb-GQD) for rapid and selective detection of Tartrazine in Food Matrices

Tharani Gomathi Ramesh[1] and Mangaiyarkarasi Rajendiran[1]*

[1]Centre for Nanotechnology Research, Vellore Institute of Technology, Vellore-632014, India

Supporting information:

2.1 Materials and Methods:

Citric acid Anhydrous was obtained from SDFCL Mumbai. Urea, Tartrazine, Sodium Citrate, and Sodium Hydroxide were purchased from Sigma Aldrich. Terbium chloride hexahydrate ($TbCl_3 \cdot 6H_2O$,99.9%) was purchased from Thermo Fisher Scientific. The chemical reagents used were of analytical grade, without any further purification. Double- deionized water was used throughout the experiment.

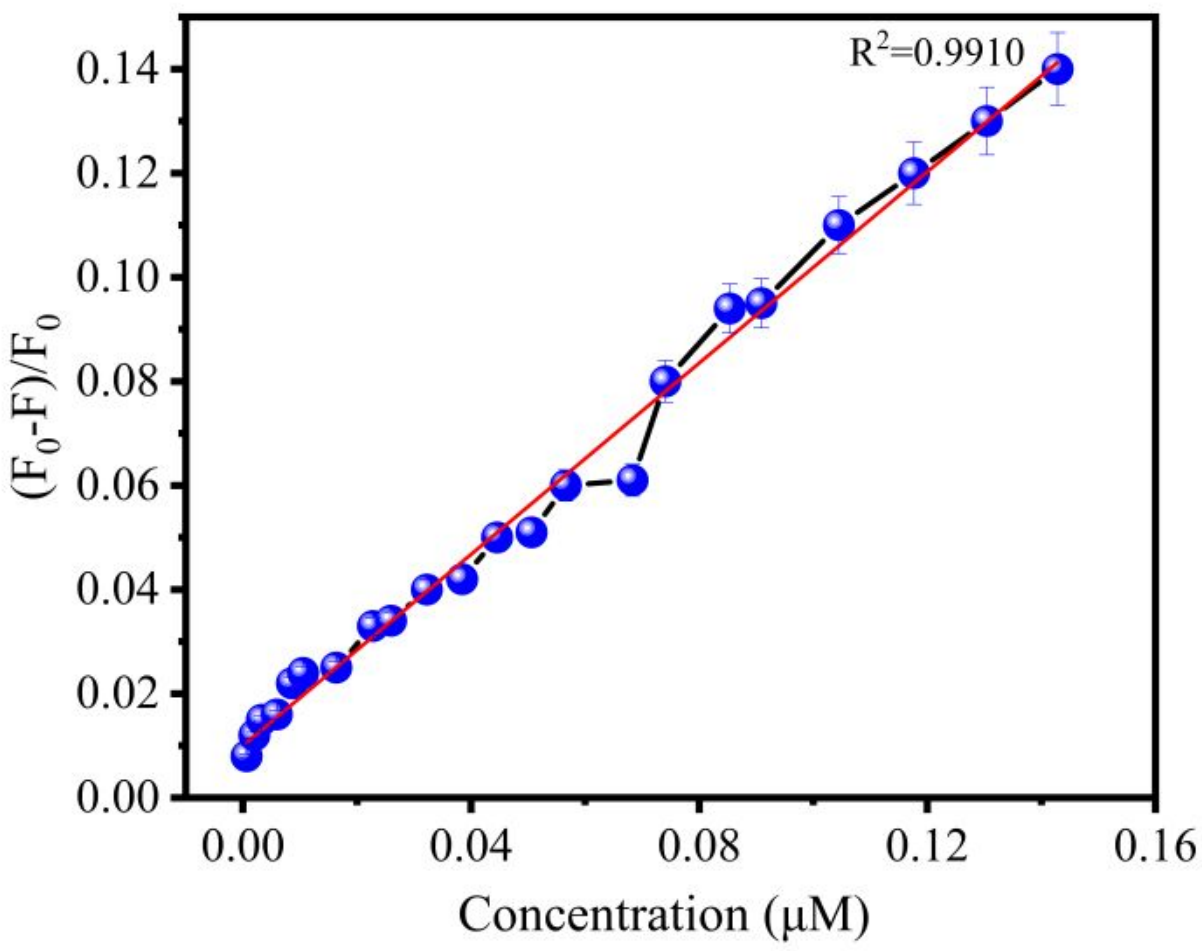

Figure S1: *Linear plot of (Fo-F)/Fo vs concentration of Tartrazine for calculating the limit of detection*.

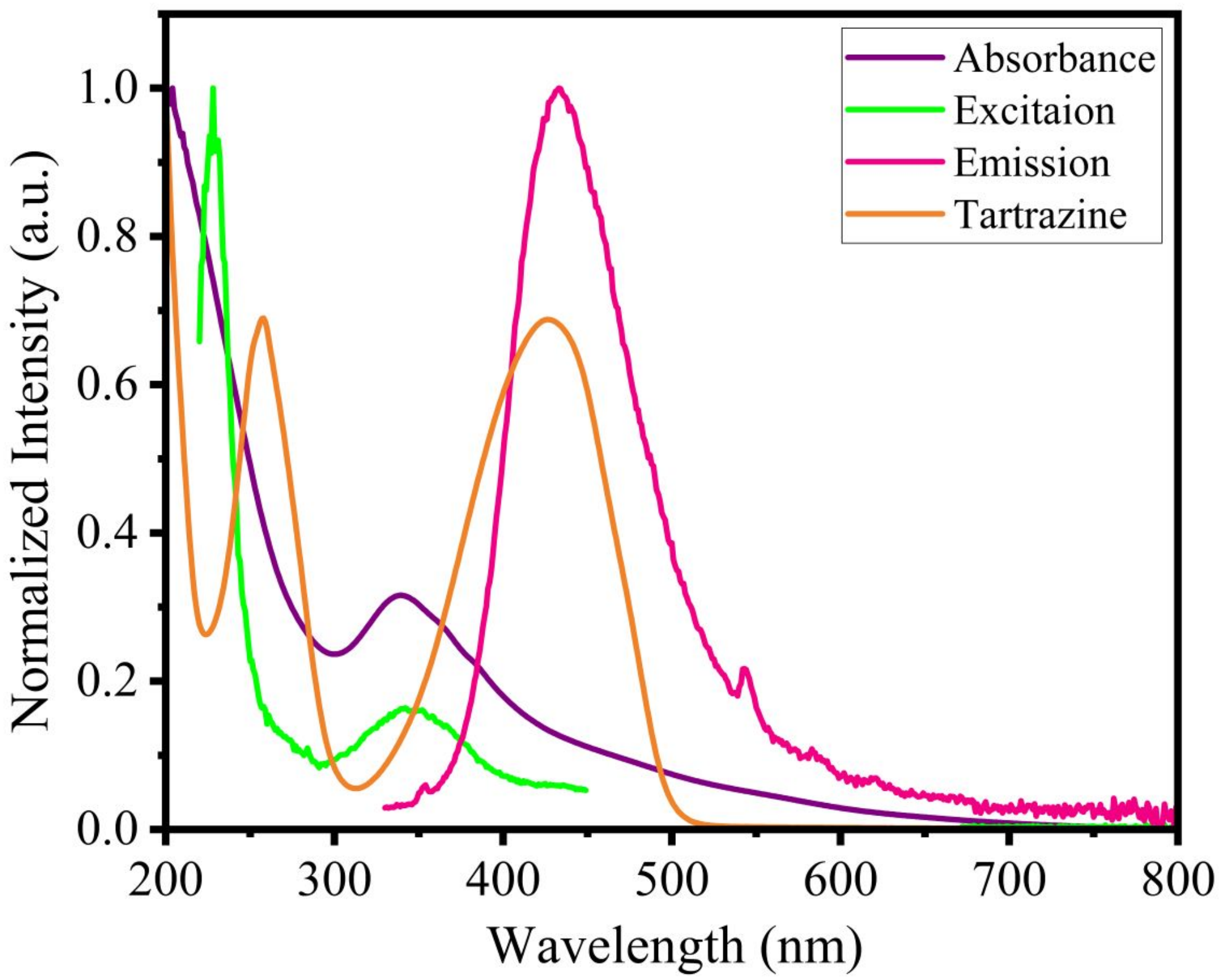

Figure S2. The absorbance spectrum of Tb-GQD, Tartrazine and the excitation and emission spectrum of Tb-GQD.

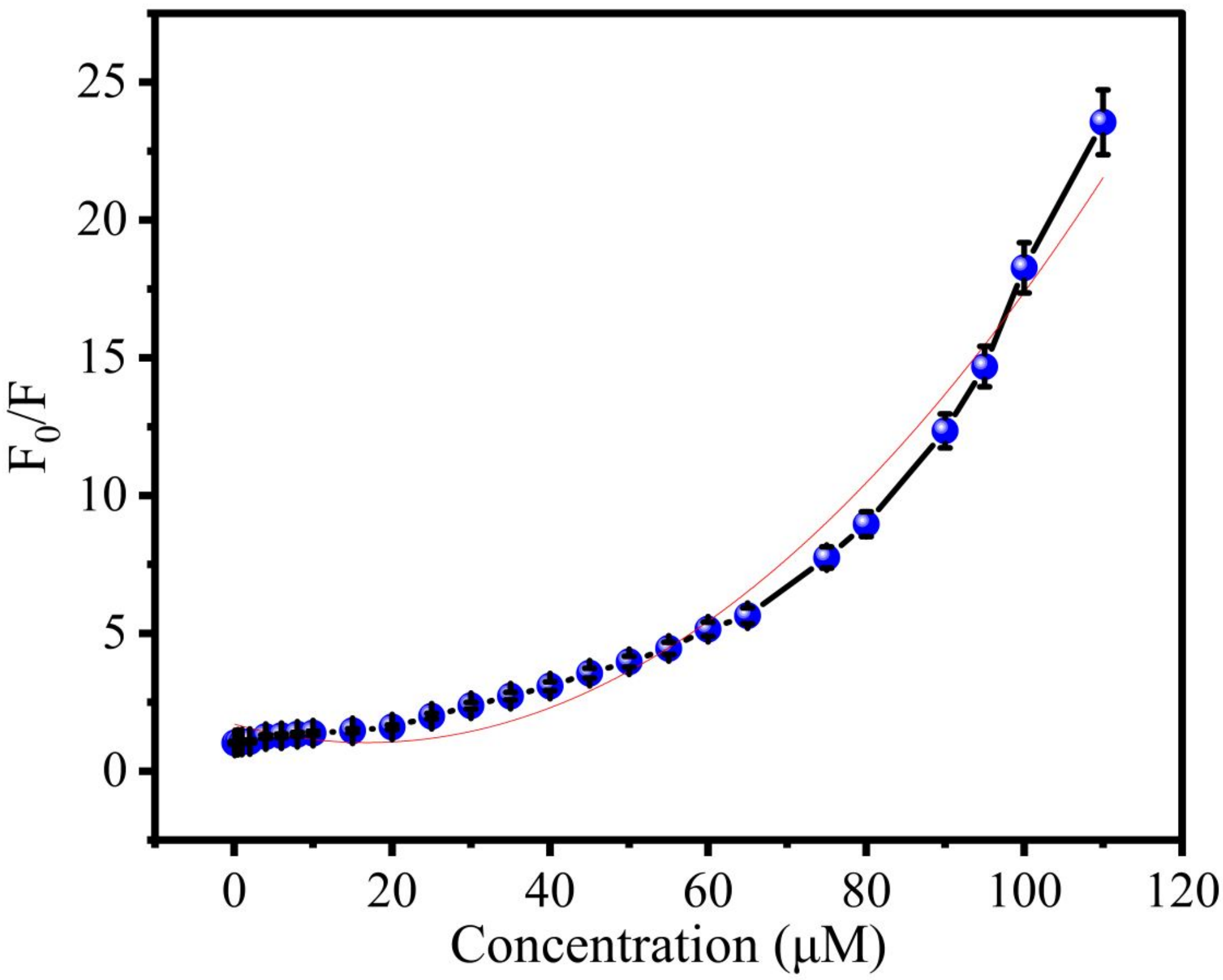

Figure S3. Stern-Volmer plot for fluorescence intensities (Fo/F) vs concentration of Tartrazine

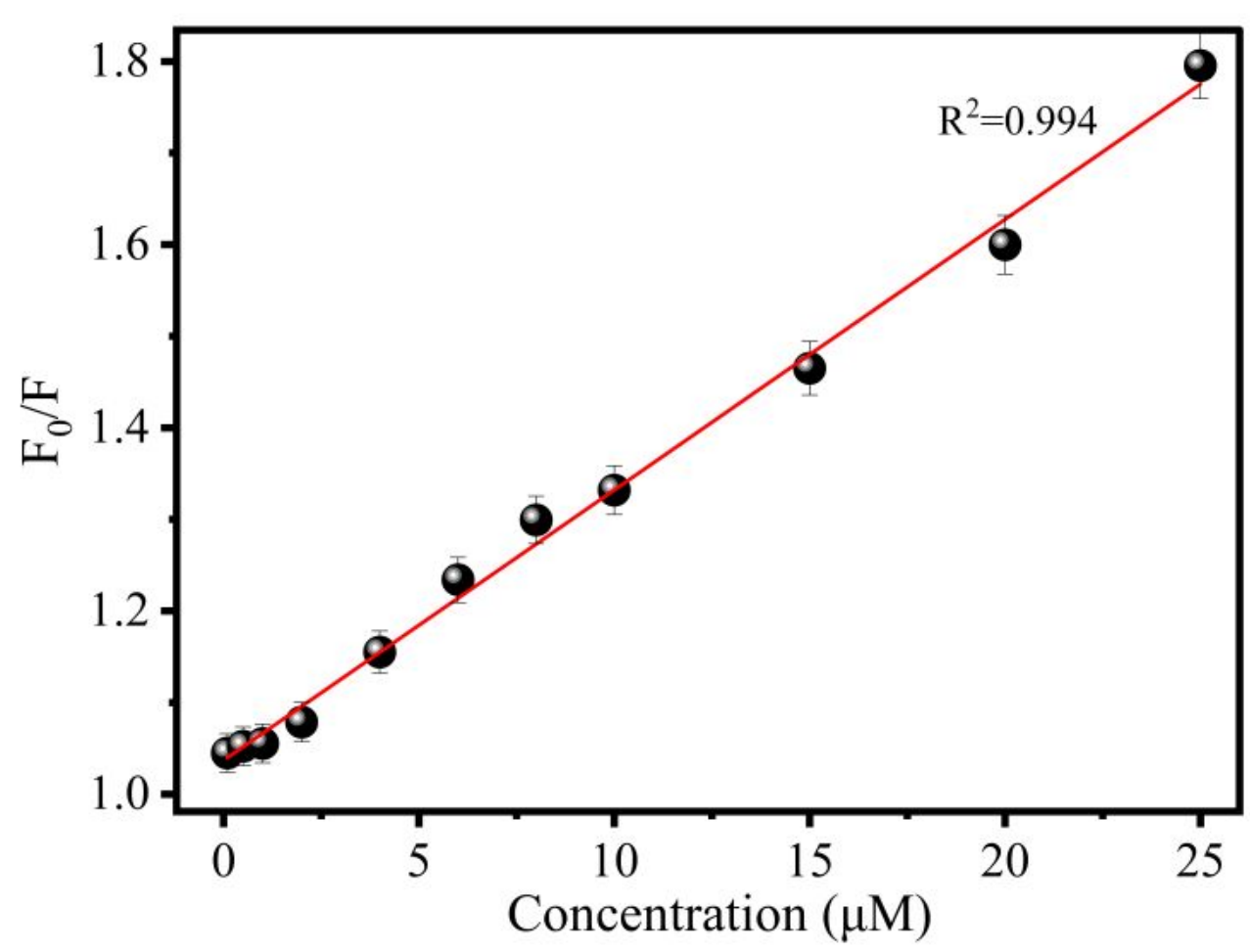

Figure S4. Stern-Volmer plot-linear plot of fluorescence intensities (Fo/F) vs Tartrazine at lower concentrations.

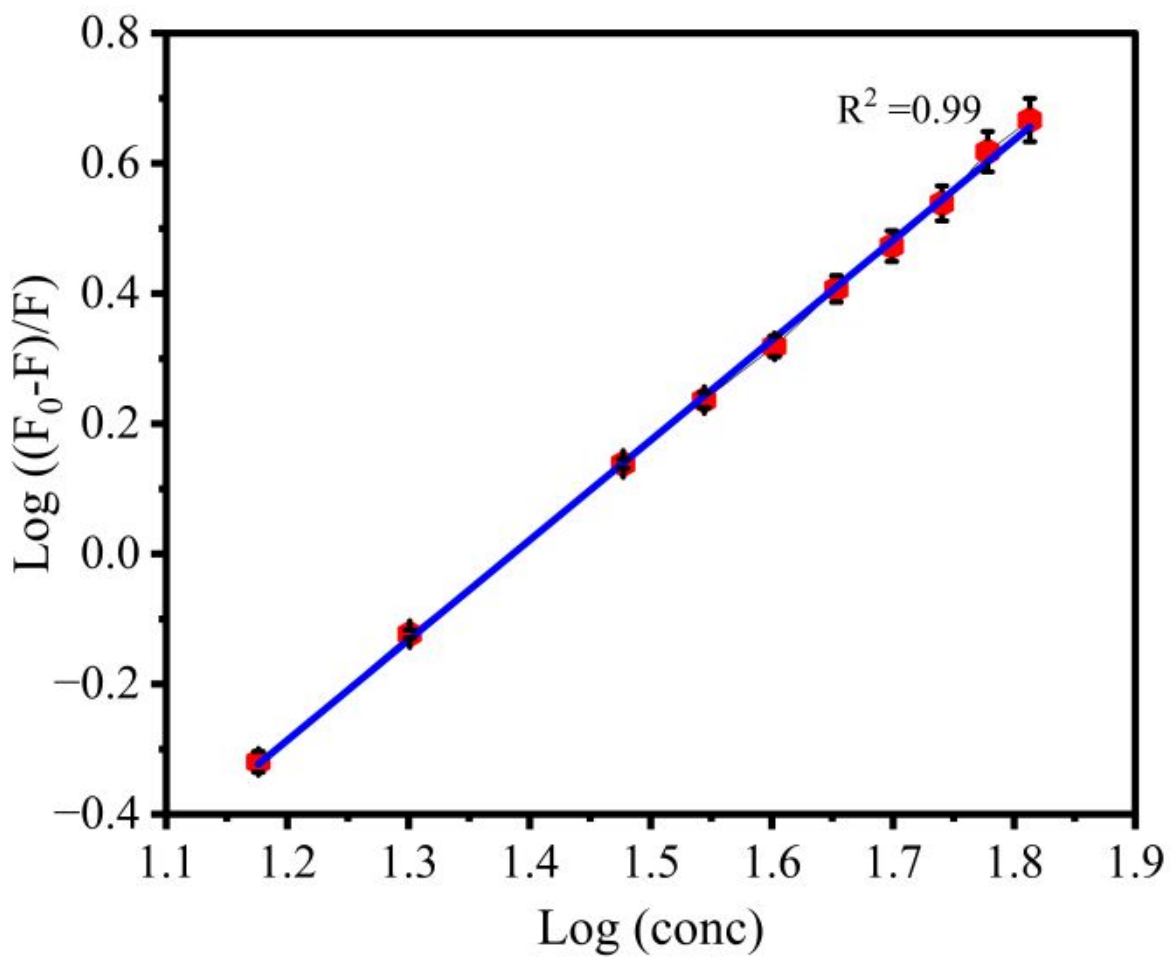

Figure S5. modified Stern-Volmer plot

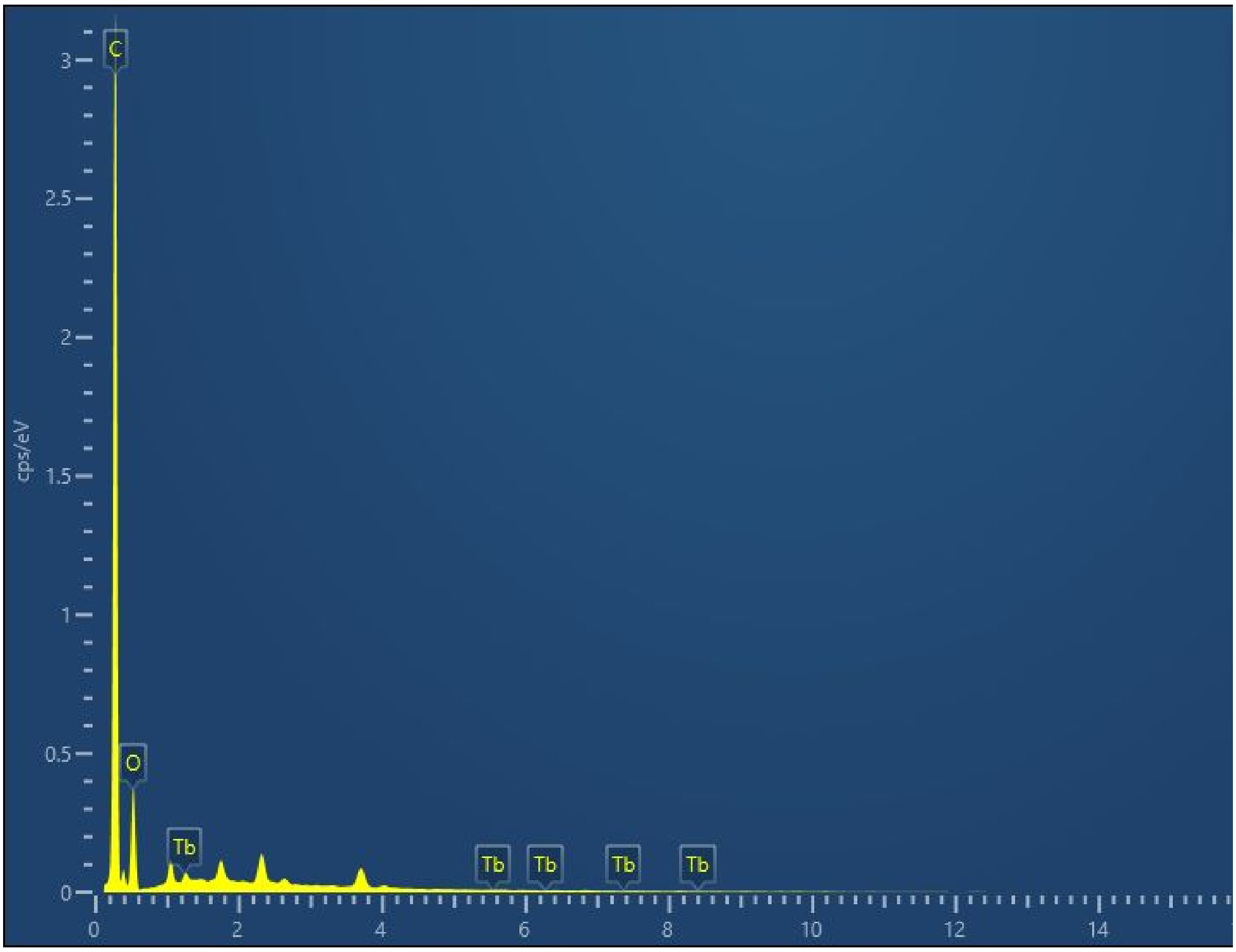

Fig S6: EDS spectrum of the Tb-GQD to confirm the presence of Terbium in GQD

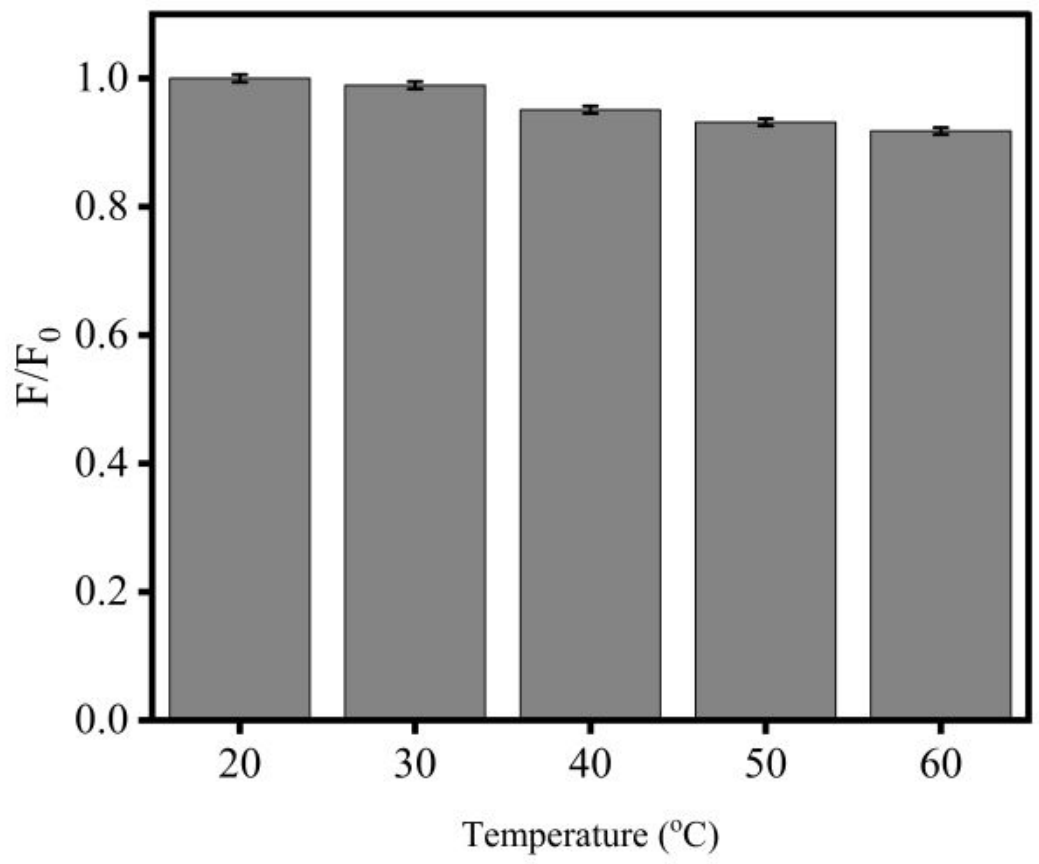

Figure S7: Temperature analysis of Tb-GQD

Table S1: Inter-day and intra-day analysis of Tb-GQDs

Day	Spiked Range (µM)	Measured Range (µM)	Recovery (%) n=3
		Inter batch	
**0th day**	10	9.73	97.3 ± 0.31
	20	19.64	98.2 ± 0.47
**1st day**		***Intra batch***	
	10	9.54	95.4 ± 0.44
	20	19.39	96.9 ± 0.27
**3rd day**	10	9.37	93.7 ± 0.29
	20	19.13	95.6 ± 0.41

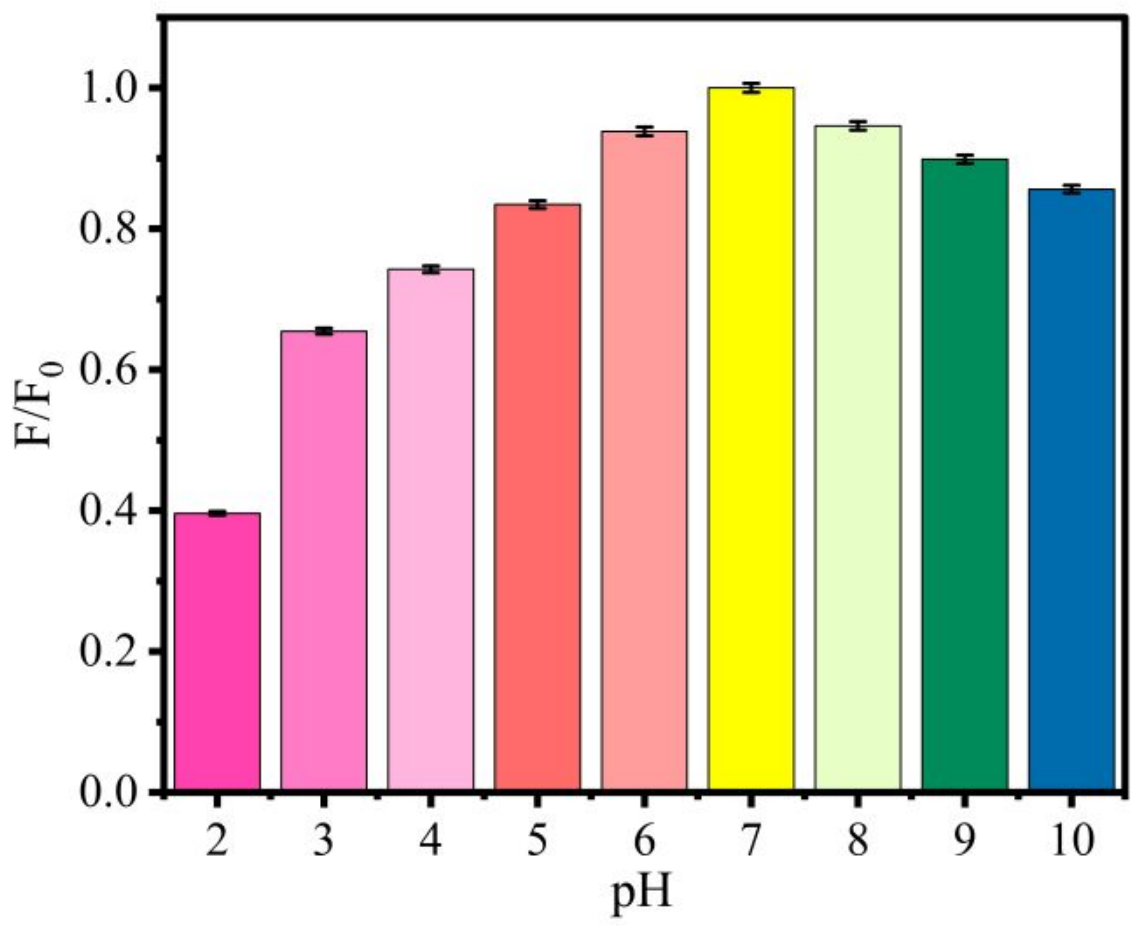

Figure S8: pH stability study of Tb-GQD
